# Near field and far field plasmonic enhancements with bilayers of different dimensions AgNPs@DLC for improved current density in silicon solar

**DOI:** 10.1038/s41598-022-22911-9

**Published:** 2022-11-16

**Authors:** Maryam Hekmat, Azizollah Shafiekhani, Mehdi Khabir

**Affiliations:** 1 Department of Nanophysics, Faculty of Physics, Alzahra University, Vanak, Tehran, 1953833511 Iran; 2 Department of Electrical Engineering, Iran University of Science and Technology, Tehran, 1684613114 Iran

**Keywords:** Materials science, Physics

## Abstract

The effect of a bilayer of different dimension silver nanoparticles (Ag NPs) on light trapping in silicon solar cells is investigated. Here, we report on the improved performance of silicon solar cells by integrating two layers of silver nanoparticles of different sizes. We experimentally examine the plasmonic near-field and far-field effects of bilayer Ag NPs embedded within an anti-reflective DLC layer on silicon solar cells' optical and electrical characteristics. Field-Emission Scanning Electron Microscopy drove the two-dimensional differences in the size of Ag NPs. The surface plasmon resonance of the two-dimensional nanoparticles was estimated from the absorption optical spectra. External quantum efficiency measurements showed that near-field or far-field plasmonic effects altered with the Ag NPs size. The development of far fields was confirmed by measuring the solar cell performance under AM 1.5 G illumination. The impact of the far-field in the cell containing two layers of Ag NPs, which outer layer is larger dimensions NPs, improves the current density up to 38.4 mA/cm^2^ (by 70% compared to the bare reference cell).

## Introduction

Renewable energy seems to be the best alternative to fossil fuels. Solar energy is the best alternative, and crystalline wafer-based silicon (C-Si) solar cells are by far the most efficient and feasible type of it^1–3^. The harness of solar energy conversion technology to electricity through the generation of electron–hole pairs in photovoltaic devices is well known. Although silicon wafers make up 85% of photovoltaic devices worldwide, the spectral response of C-Si solar cells is low in wavelengths less than 450 nm and above 1100 nm^4–6^.

The fundamental factor limiting the conversion efficiency is the spectral mismatch between C-Si solar cells and the incident radiation. C-Si solar cells' maximum theoretical conversion efficiency is approximately 30%^7^. High reflection and rapid combination of high-energy photons also limit efficiency. The most critical challenge in comprehensively replacing solar cells is achieving acceptable efficiencies. Recently, it has been keenly focusing on applying anti-reflective layers and metal nanoparticles (NPs) on the surface to reduce light reflection, which leads to increased light harvesting. In particular, researchers are employing silver nanoparticles (Ag NPs) and gold (Au NPs) to enhance light trapping performance. Metal nanoparticles improve the solar cell's performance via enhancing light harvesting scattering and electron carriers from the high electric field by localized surface plasmonic resonance (LSPR) coupling with incident light^8,9^. Carbon-based materials could be appropriate choices for anti-reflective applications due to their environmental and easy production on a large scale in solar panels. Diamond-like carbon (DLC) is one of the most well-known carbon structures with an amorphous carbon structure with sp^2^ and sp^3^ sites. Properties similar to diamonds include high thermal conductivity, hardness, anti-reflective, high electrical resistivity, and tunable bandgap by manipulating sp^2^ and sp^3^ bonding ratio^10^. The embedded Ag in the DLC lattice prevents the agglomerated nanoparticles and the growth of large and clustered nanoparticles. Our previous reports have shown that the effect of gold and silver nanoparticles in the DLC lattice has led to increased efficiencies in quantum dot solar cells^11,12^.

Plasmonic excitation to increase solar cell performance is well known. Although several studies have concentrated on a single plasmonic layer, it has been proven that applying two layers is more efficient^13,14^. Nevertheless, the compound of Ag NPs in the lattice of an anti-reflective structure in C-Si solar cells has rarely been reported^9^.

This study investigated the plasmonic and anti-reflected simultaneous effects of new structure Ag NPs@DLC. In most studies of multilayer Ag NPs, particles of uniform size are considered while we report two layers of Ag NPs @DLC employed with non-uniform dimensions on the surface. A new style of non-uniform bilayer decorated with silver nanoparticles on the p–n junction is increased light trapping and improves solar cell characteristics. The overall short-circuit current density (Jsc) and power conversion efficiency (PCE) has been obtained at 38.4 and 4.98%, an increase of 70% and 48%, respectively, in our proposed structure.

## Experimental details

To fabricate plain C-Si solar cells, p-type single-crystalline Si (100) wafers measuring 15 mm × 15 mm with a thickness of 250 nm and resistivity > 120 Ω-cm were polished preferentially. Samples were cleaned with H_2_O_2_:H_2_SO_4_ (1:3), Acton, rinsed with de-ionized water for 60, 15, and 15 min, respectively, and then dried with nitrogen gas. Subsequently, the wafers are then etched in a 5% HF solution for 2 min to remove native oxide. To form the p–n junction, wafers with a flat surface were n-doped by phosphoryl chloride diffusion. The wafers were first injected with nitrogen gas for 30 min in an open tube furnace at 900 °C and afterward objected to phosphorus diffusion for 10 min. Followed Al deposition by RF-sputtering with Al target to form the back and front pattern contact surface. In the next step, the surface of photoanodes is decorated with Ag NPs embedded in the DLC network by co-deposition of RF-sputtering and RF-PECVD method. Sputtering is one of the most popular methods of physical vapor deposition (PVD) for uniform deposition of plasmonic nanoparticles on a surface. The sputtering process was performed with a 99.9% purity silver target with acetylene gas. The chamber working pressure was maintained at 13.5 mTorr with acetylene gas; for the first step-deposition was performed with smaller nanoparticles with RF power of 50 W, and a deposition time of 1 min was used. The second step sets the RF power to 80 W to achieve larger nanoparticles. Finally, the wafers were annealed at around 300 °C to eliminate possible contaminations and form uniform round nanoparticles^15,16^. To prevent the negative effect of temperature on solar cell parameters, nanoparticles were annealed at 300 °C^17^. Annealing of the layers leads to the formation of Ag NPs and the reduction of density on the substrate.

**Figure 1:**
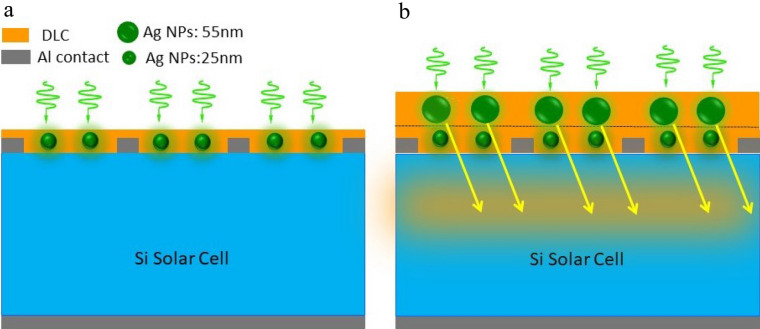
Schematic representation of Ag NPs embedded at DLC: (**a**) monolayer and (**b**) bilayer which create different plasmonic light-trapping.

A schematic of configurations of different plasmonic structures for light trapping is shown in Fig. 1. The plasmonic near-field coupled with the active layer when Ag NPs are on top of the surface layer of the semiconductor surface and increases the absorption in the around region of nanoparticles as shown in Fig. 1a. In the second layer, large Ag NPs are placed far from the cell's active layer in which the LSPR is created in far fields, which strongly scatter light into the active layer (Fig. 1b). This is a cost-effective and reproducible technique for photovoltaic applications^18^. The interaction between the electromagnetic field of the incident light and the surface charge of the bigger size plasmonic nanoparticles increases the electric near and far-field, enhancing the light absorption and the solar cell efficiency.

Eventually, morphology, optical, and performance of solar cells were measured. The morphology surface p-n/Ag@DLC was obtained by field emission scanning electron microscopy (FESEM- MIRA III- TESCAN). A grazing X-ray Diffractometer having the Cu Kα source (XRD- PHILIPS- PW1730) was employed to determine the structural change in the Ag NPs. Absorption properties of different surfaces were studied by UV–Visible absorption spectroscopy (PhyTec, MA-2500). The photocurrent density–voltage (J–V) characteristics of solar cells were recorded under sun illumination (AM1.5 G, 100 mW/cm^2^) using a solar simulator (Sharif Solar- PGE-18). Incident light to Current Efficiency (IPCE) spectra was recorded using an IPCE measurement system (Sharif Solar- IPCE-020).

## Result and discussion

### Morphology

**Figure 2:**
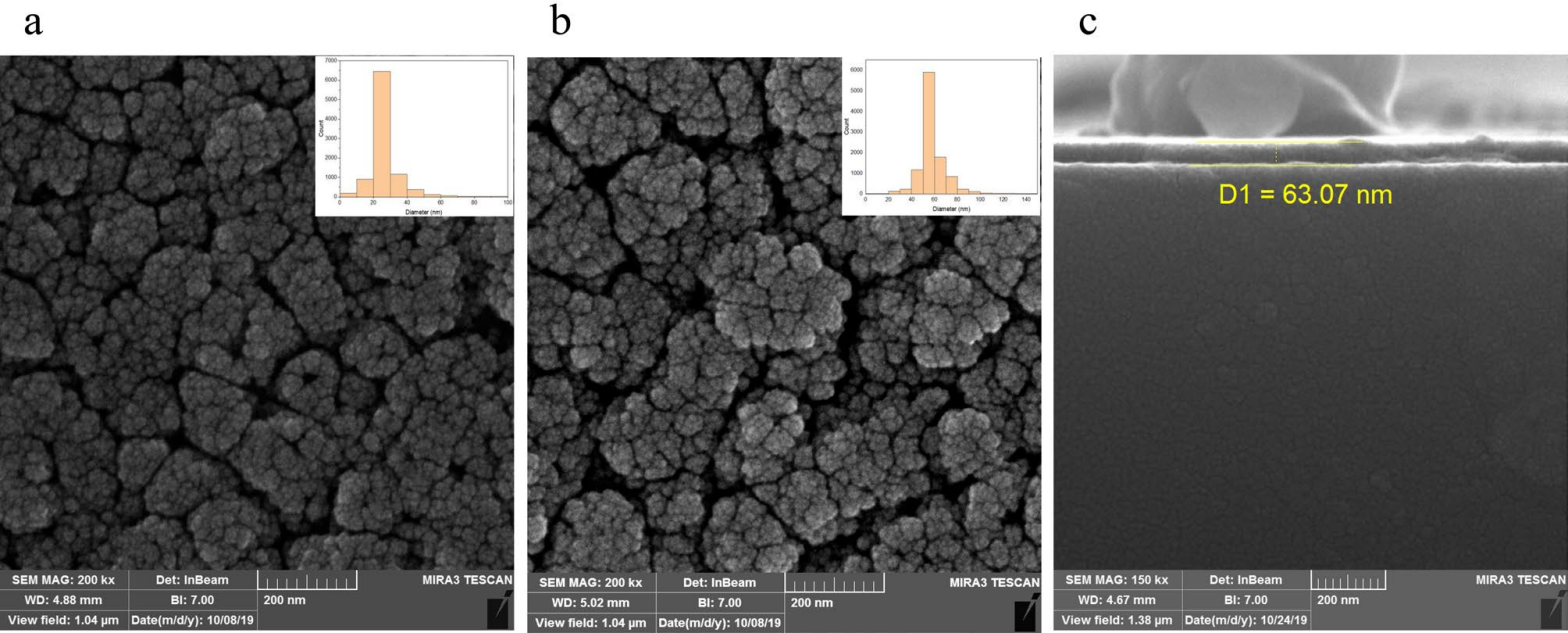
Top view of FESEM image of Ag NPs in (**a**) first layer, (**b**) second layer, (**c**) cross section of DLC layer.

Field emission scanning electron microscopy (FESEM) of Ag NPs for monolayer and bilayer are shown in Fig. 2a,b, respectively. The Ag NPs ranged from 25 nm with an average diameter of 27 nm for the monolayer and 56 nm for the bilayer. A significant change in the size of silver nanoparticles is observed in the sample with the second layer of silver. These larger particles in the second layer are the reason for the formation of far-field LSPR. Figure 2c shows a cross-section of the DLC layer when deposited on the surface by the RF-PECVD method.

**Figure 3:**
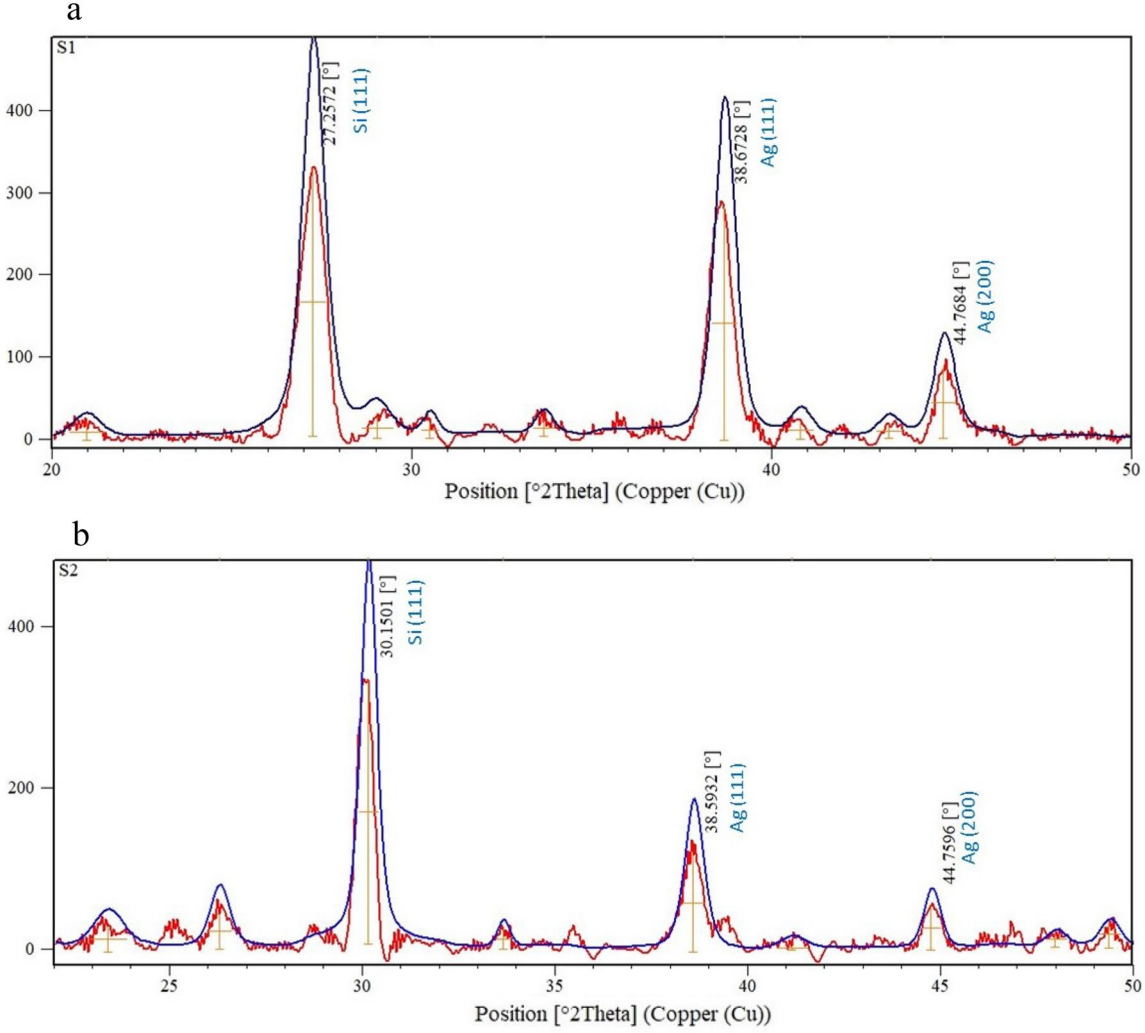
XRD diffraction of the monolayer and bilayer different dimensional of Ag NPs.

The XRD pattern of monolayer and bilayer Ag NPs cells is shown in Fig. 3. It's seen that Ag NPs show two majors diffraction peaks at 38.5° and 44.7° and the central diffraction peak around 27° to 30° is related to Si. The right shift in diffraction peak for Si is attributed to the surface effect of NPs^19^. The observed peaks index was found to be (111) and (200) for Ag NPs and (111) for Si, respectively, for the observed angles. The results confirm that the Ag NPs and the peaks matched the reported JCPDS card number 87-0597^20^. Also, XRD diffraction of the two samples confirms that nanoparticle size increased in the bilayer Ag NPs sample. The full-width half maximum (FWHM) in major diffraction peak at 38.5° decreased from 0.68 in the sample containing a monolayer of nanoparticles to 0.55 in the second sample. According to Debye–Scherrer's formula, the decrease in FWHM is equivalent to an increase in the nanoparticle diameter of the proposed bilayer Ag NPs^21^.

### Optical properties

**Figure 4:**
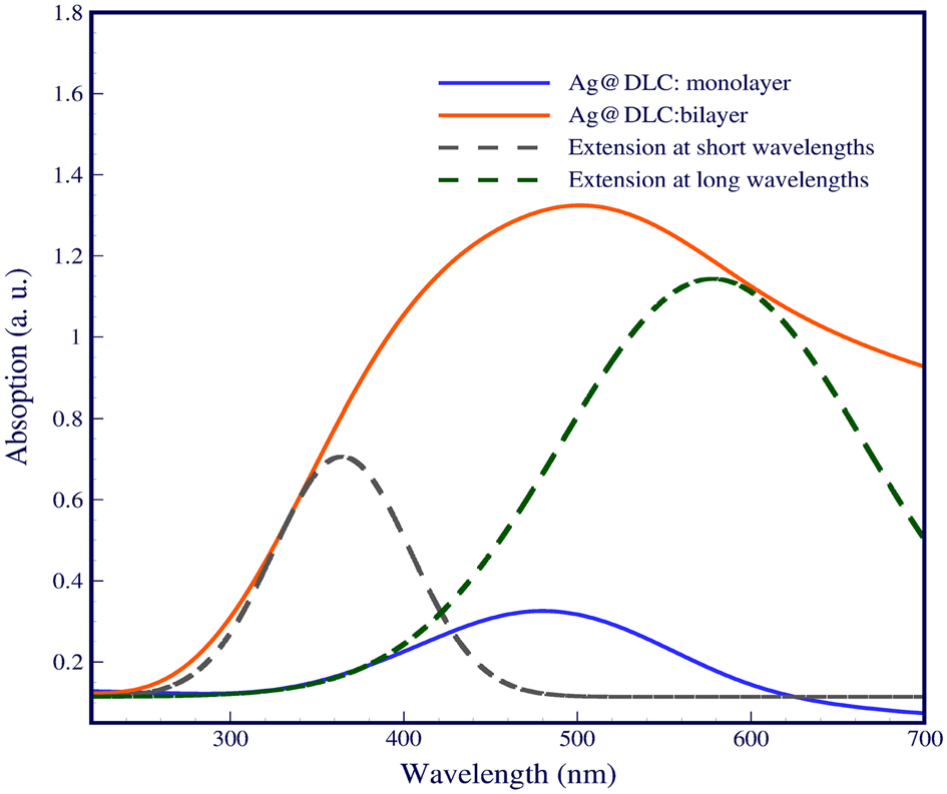
Normalized absorption spectra of the monolayer and bilayer different dimensional of Ag NPs.

As Fig. 4 shows, by decorating the silicon surface with Ag NPs, the absorption increases in the ultraviolet and visible range because of the LSPR effect. As shown in Fig. 4, a bilayer decorated with Ag NPs with different radii indicates more absorption enhancement than a decorated monolayer of nanoparticles. The noticeable increment can be seen in the dimensions of bilayer Ag NPs compared to monolayers. The interaction of the light with the small Ag NPs increases the localized plasmonic due to near field enhancement and causes to increase in adsorption. The increase in absorption at λ < 400 nm is attributed to near fields. When large Ag NPs are placed in front of the cell in the interface between the active layer and air, far-field scattering shows itself obviously. The proposed structure of large Ag NPs/small Ag NPs, not only confirms that the nanoparticle size is a critical factor in localized surface plasmonic resonance but also increases absorption at λ > 550 nm in this sample resulting from enhancement in the far-field.

Near and far-field are multiple plasmonic enhancement mechanisms in the proposed structure has occurred in parallel and caused high absorption in short and long wavelengths. In addition, materials with higher permeability are preferable to light^22^, and then DLC thin film scatters light towards the active layer and causes high absorption. On the other hand, breaking the symmetry by changing the size of silver nanoparticles has improved the absorption performance. In gold and silver nanoparticles, plasmons can be excited in the range of visible frequencies.

### Electronic properties

**Figure 5:**
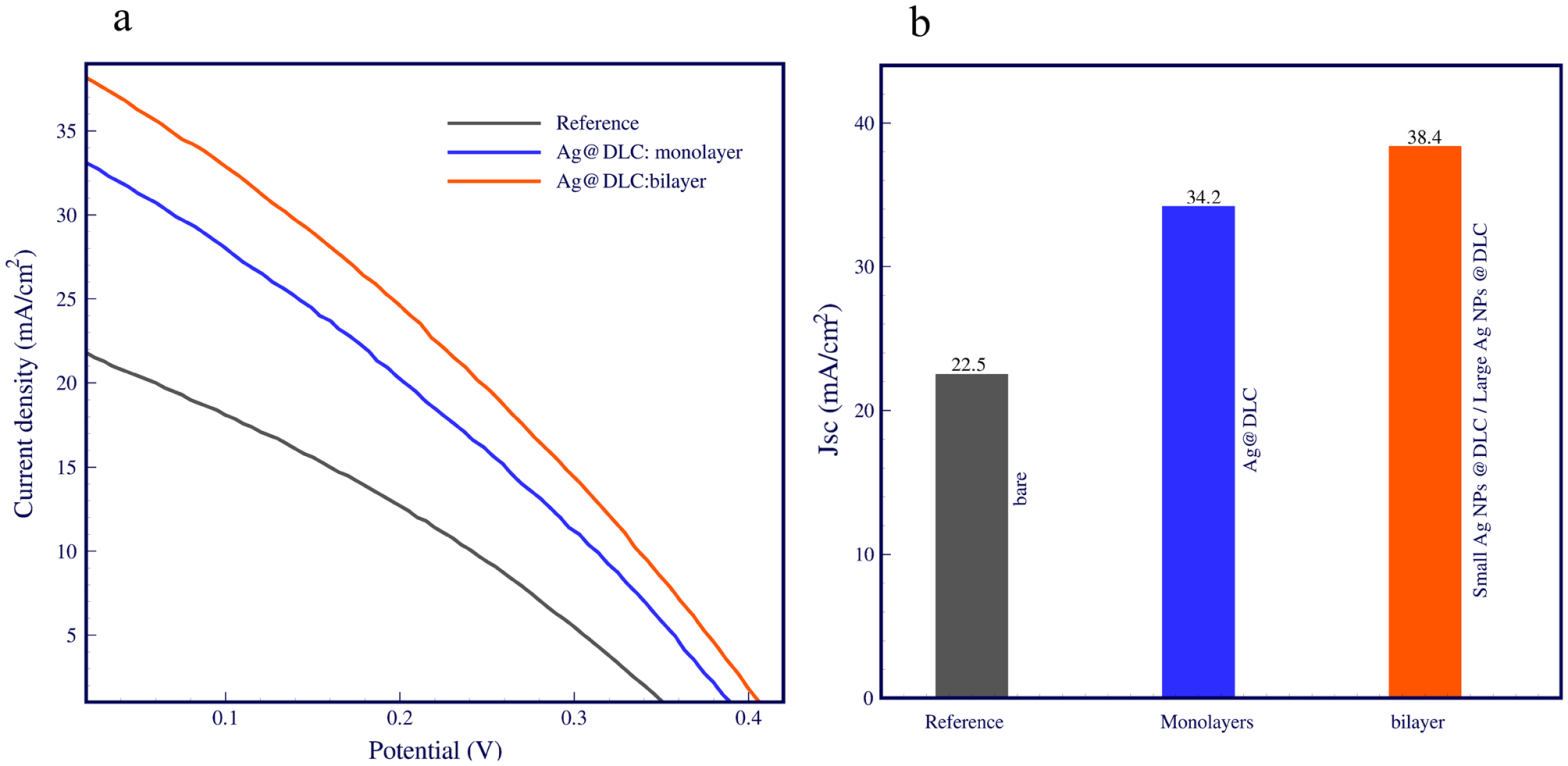
(**a**) Photovoltaic J–V curves of bare cell, monolayer and bilayer Ag nanoparticles solar cells under AM 1.5 solar radiation, (**b**) comparison of the Jsc.

Figure 5a shows J-V curves of cells, and detailed photovoltaic parameters are summarised in Table 1. The result demonstrates that decorated monolayer Ag NPs improve PEC, confirming the LSPR effect of Ag nanoparticles enhanced light harvesting. Increased short-circuit currents with Au and Ag nanoparticles used on top of silicon solar cells have been reported in many studies^23,24^. Figure 5b illustrates the J_sc_ for the bare reference cell, the solar cell with a monolayer of approximately 30 nm of Ag NPs, and the solar cell with a bilayer of Ag NPs, the last layer containing larger particles. Open circuit voltage of 360 mV, short circuit current density of 22.5 mA/cm^2^, and power conversion efficiency of 2.54% were obtained in the bare solar cell as reference values. Using metal nanoparticles in similar studies, short-circuit currents of up to 45%^25^ were reported. Our study achieved a 52% enhancement in short-circuit current (34.2 mA/cm^2^) compared to bare cells by containing a layer of Ag@DLC due to Ag plasmonic effect. The results indicate a 70% exceed of bare cell in short-circuit current in a cell with bilayer's different dimensional Ag NPs. Plasmonic resonance in far-field raises short-circuits current in 38.4 mA/cm^2^.

**Table 1:** Photovoltaic performances of all solar cells.

Sample	V_OC_	Jsc (mA/cm^2^)	PCE%	FF%
Reference: bare cell	360	22.5	2.54	31.4
Cell with a monolayer of Ag@DLC	396	34.2	4.08	30.1
Cell with bilayer's different dimensional Ag@DLC	407	38.4	4.98	31.9

Also, Ag NPs are placed in the DLC matrix to prevent agglomeration and forming clusters, which is direct light to the cell. The tendency of light towards high permeability materials is one of the positive effects of DLC. Coating the Si surface with a layer of DLC and embedding Ag NPs prevents the formation of Ag recombination centers. In addition, one of the reported effects of the DLC layer is to increase cell stability and further improve efficiency by ultraviolet irradiation^26^.

**Figure 6:**
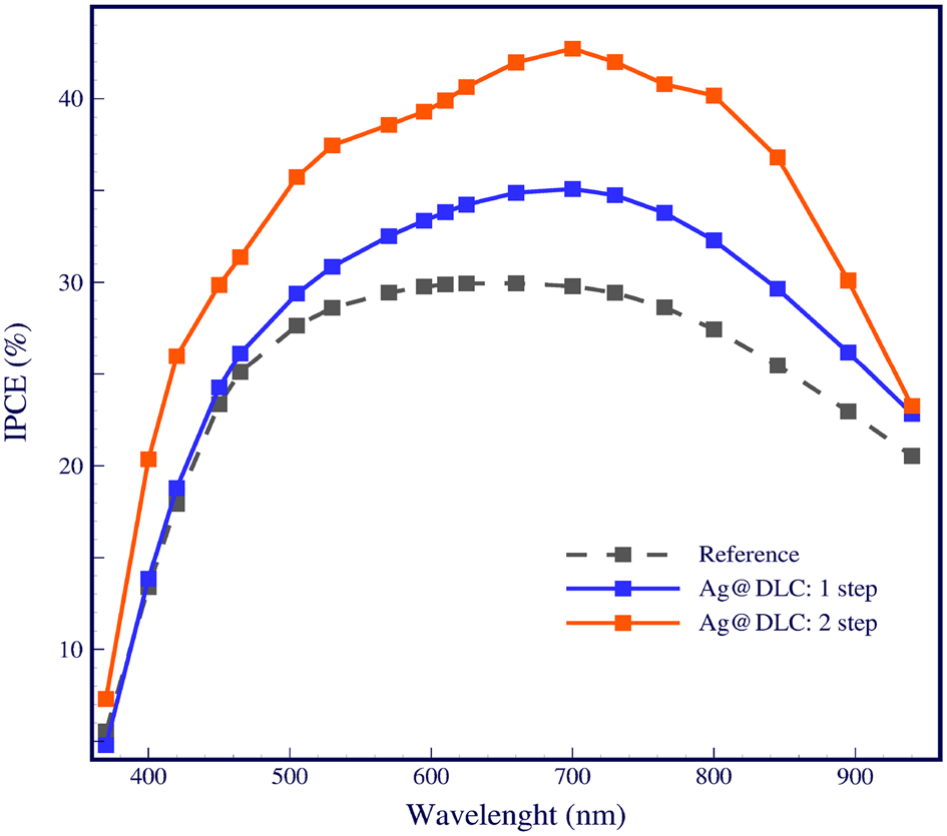
Incident photon to current efficiency (IPCE) spectra of the bare cell, monolayer and bilayer Ag nanoparticles solar cells.

Figure 6 is compared the normalized result of incident photon to current efficiency (IPCE), referred to as external quantum efficiency (EQE) spectra. It is seen that the EQE is significantly improved over that of the reference cell. The EQE values agreed with the optical absorption values of bare cells and Ag decorated cells. As the IPCE curves significate the increased values across the entire wavelength range (370–940 nm) can be attributed to the plasmonic absorption and near field scattering of Ag NPs in monolayer Ag@DLC and far-field scattering of Ag NPs in bilayer Ag@DLC as well as the anti-reflective effects of the DLC layer. The IPCE values confirm that the light trapping was enhanced by bilayer different dimensions of Ag NPs LSPR.

To understand the increase in current, we must look at the changes in the fields. As demonstrated in Fig. 1, the generation of the field around the silver nanoparticles is localized and of the near field type. In Ag NPs bilayers, far fields became more robust, making it accessible to couple light into the active layer. As the figure shows proposed sample enhanced the light absorption. Because the increase in nanoparticle size leads to plasmonic resonance redshift absorption at longer wavelengths, using two different sizes, nanoparticles have synergy in absorbing at short and long wavelengths and eventually lead to a decreased light loss in the cell.

Fundamental interpretation of light coupling with LSPR, which is a function of the change in the size of Ag NPs, refers to Mie scattering theory^27^, which makes it possible to describe the plane monochromatic wave scattering by a homogeneous sphere surrounded by a homogeneous medium for particle radius and any material. The optic effect in scattering is attributed to the light response to an external electric field, and this is due to the inherent characteristic of a spherical conductive nanostructure embedded in a dielectric environment.

The function of metal NPs in increasing the optical efficiency of solar cells can be explained for two reasons. First, the surface plasmon energy generated is emitted as light and scattered in the active area, which leads to an increase in the optical path length in the silicon and more photons being absorbed. Second, excitation of the surface plasmons increases the localized electric field and absorption due to the high density of the photon states^28^.

## Conclusion

This study experimentally examined the plasmon coupling (near-field and far-field) of two-dimensional Ag NPs deposited over a silicon solar cell embedded within an anti-reflective DLC layer. Substantial local field increase around metal nanoparticles caused by LSPR can effectively use in thin-film solar cells. When small metal nanoparticles would embed in the anti-reflective material, it acts as an optical antenna for the incident light, which stores the incident energy in LSPR. Our study results of plasmon resonances are a successful example that applied the appropriate particle size proposed in theoretical studies in solar cells.

In summary, we have proposed and evaluated through morphology, optic, and electronic C-Si wafer decorated with bilayer different dimensional Ag NPs. Ag nanoparticles with different dimensions were embedded in DLC thin film net on a C-Si substrate to compare the photovoltaic performance of monolayer Ag@DLC and bilayer Ag@DLC. Optical absorption spectra showed significantly increased light-harvesting when the surface was designed with two layers of different sizes of Ag NPs. The surface plasmon effect of silver nanoparticles in bilayer Ag@DLC PV structure showed an improved cell performance with enhanced short-circuit current over monolayer Ag@DLC PV structure. In the proposed cell, short-circuit current is obtained at 38.4 mA/cm^2^, and the efficiency is 4.98%, which is 70% and 48% higher than the reference cell, respectively. Our work provides an in-depth understanding of the light absorption enhancement mechanism in the front layers of cells with Ag nanoparticles of different sizes which would yield more efficient plasmonic NPs-based solar cells.

## Data Availability

All data generated or analyzed during this study are available from the corresponding author upon reasonable request.
